# Vinculin Y822 is an important determinant of ligand binding

**DOI:** 10.1242/jcs.260104

**Published:** 2023-06-14

**Authors:** Gillian DeWane, Nicholas M. Cronin, Logan W. Dawson, Christy Heidema, Kris A. DeMali

**Affiliations:** ^1^ Department of Biochemistry and Molecular Biology, University of Iowa, Iowa City, IA 52242, USA; ^2^ Molecular Medicine Graduate Program, Roy J. and Lucille A. Carver College of Medicine, University of Iowa, Iowa City, IA 52242, USA

**Keywords:** Vinculin, Mechanotransduction, Cell–cell adhesion, Cell–matrix adhesion

## Abstract

Vinculin is an actin-binding protein present at cell–matrix and cell–cell adhesions, which plays a critical role in bearing force experienced by cells and dissipating it onto the cytoskeleton. Recently, we identified a key tyrosine residue, Y822, whose phosphorylation plays a critical role in force transmission at cell–cell adhesions. The role of Y822 in human cancer remains unknown, even though Y822 is mutated to Y822C in uterine cancers. Here, we investigated the effect of this amino acid substitution and that of a phosphodeficient Y822F vinculin in cancer cells. We observed that the presence of the Y822C mutation led to cells that proliferate and migrate more rapidly and contained smaller focal adhesions when compared to cells with wild-type vinculin. In contrast, the presence of the Y822F mutation led to highly spread cells with larger focal adhesions and increased contractility. Furthermore, we provide evidence that Y822C vinculin forms a disulfide bond with paxillin, accounting for some of the elevated phosphorylated paxillin recruitment. Taken together, these data suggest that vinculin Y822 modulates the recruitment of ligands.

## Teaser

**Summary:** Vinculin Y822 modulates ligand binding to control focal adhesion morphology and tumorigenicity in cancer cells.

## INTRODUCTION

All cells in the body encounter mechanical forces throughout their lifetimes. Cells experience many types of forces, including fluid shear stress, stretching, tension and compression (Chanet and Martin, 2014; Tschumperlin, 2011). Cells sense mechanical stimuli via membrane-bound adhesion receptors. Epithelial (E-)cadherin is one such receptor. E-cadherin forms homophilic dimers with cadherins on neighboring cells to support cell–cell adhesion. Integrins are another type of adhesion receptor; these bind to components of the extracellular matrix (Chanet and Martin, 2014; Leckband and de Rooij, 2014; Leerberg and Yap, 2013; Sun et al., 2016; Tschumperlin, 2011). Upon sensing force, integrins and cadherins recruit and activate numerous signaling partners. RhoA stimulates a signal transduction pathway that, in turn, promotes actomyosin contractility by stimulating actin polymerization and bundling and activating non-muscle myosin II. The net result is reinforcement of the actin cytoskeleton and growth of the adhesion complexes – two events critical for withstanding force (Lee et al., 2019a; Matsushita et al., 2011; Shimozawa and Ishiwata, 2009; Uyeda et al., 2011).

In response to force, the protein vinculin is recruited to integrins and cadherins. Vinculin is comprised of eight anti-parallel α-helical bundles organized into five distinct domains (Borgon et al., 2004; Winkler et al., 1996; Ziegler et al., 2006). Domains 1–4 (D1–4) form a head that is connected to a tail domain (D5) via a short linker. Vinculin exists in at least two conformations – an open extended conformation, and a closed conformation where the head and tail domains engage in intramolecular interactions that are unfolded upon exposure to force (Bakolitsa et al., 2004; Chen et al., 2006; Chorev et al., 2018; Cohen et al., 2006; del Rio et al., 2009; Sun et al., 2017; Wang et al., 2021; Yao et al., 2015). These conformational changes expose binding sites for ligands (Gilmore and Burridge, 1996; Golji and Mofrad, 2010; Johnson and Craig, 1994, 1995a,b; Stec and Stec, 2022). Several proteins bind to the vinculin head domains including talin, IpaA, α-catenin and β-catenin proteins. Other molecules, such as paxillin, phospholipids and actin bind to the tail domain (Brown et al., 1996; Golji and Mofrad, 2013; Johnson and Craig, 1995a; Menkel et al., 1994; Turner et al., 1990; Wood et al., 1994). Vinculin binding to actin is thought to be especially important, as it provides a mechanism for force to be transmitted to the actin cytoskeleton (Goldmann, 2016; le Duc et al., 2010).

Vinculin function is modulated in part by phosphorylation at specific residues (Auernheimer and Goldmann, 2014; Auernheimer et al., 2015; Bays et al., 2014; Golji and Mofrad, 2010; Golji et al., 2012; Küpper et al., 2010). In fact, tyrosine phosphorylation of vinculin at Y822 is critical for E-cadherin to transmit force to the cell interior. Indeed phosphodeficient Y822F vinculins are unable to reinforce the actin cytoskeleton in response to increased tension in normal mammary epithelial cells (Bays et al., 2014). Other work has demonstrated that cells expressing Y822F vinculin have increased survival owing to increased recruitment of focal adhesion kinase (FAK; also known as PTK2) to paxillin, a vinculin-binding partner (Subauste et al., 2004). This evidence suggests that Y822 regulates the biological response of cells. Although Y822 has these critical functions, its role in disease remains largely unexplored. An examination of The Cancer Genome Atlas Database has revealed that Y822 is mutated to cysteine in some individuals with uterine cancer (https://portal.gdc.cancer.gov/genes/ENSG00000035403).

Here, we explore the role Y822F and Y822C amino acid substitutions have on vinculin biology. Interestingly, a Y822F vinculin substitution produces contractile cells that have larger numerous focal adhesions that support migration and proliferation. In contrast, cells expressing Y822C substitutions produce cells that are less well spread and have smaller, but more numerous focal adhesions. However, these cells grow and migrate faster than cells expressing wild-type vinculin. We investigated the mechanisms for these effects and found that the two mutants differentially bind to several vinculin-binding partners. Most notably, vinculin Y822C binds paxillin but not talin (herein referring collectively to talin 1 and 2) robustly. In contrast, Y822F binds more talin but binds paxillin less well. The increased binding was abrogated by the addition of N-acetyl-cysteine (NAC) or by mutating Y822 to Y822A or Y822S, suggesting that disulfide bond formation is critical for increased paxillin binding. This work provides a novel mechanism by which different mutations at vinculin Y822 can affect focal adhesion biology and recruitment of ligands.

## RESULTS

### 4T1 cells that lack vinculin show altered morphology

**Fig. 1.**
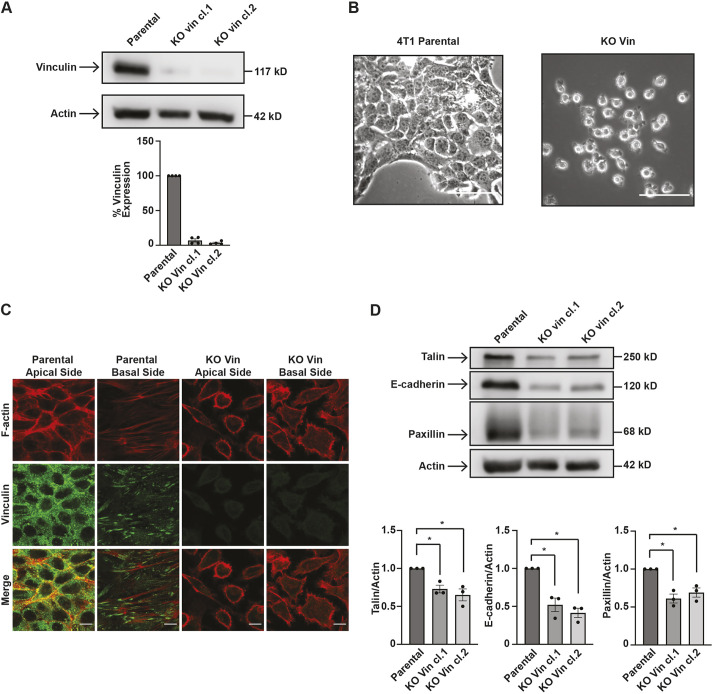
**4T1 cells lacking vinculin have altered phenotypes.** (A) Vinculin expression was stably inhibited using CRISPR. Two guide RNAs targeting mouse vinculin were designed and inserted into a plasmid containing Cas9. 4T1 cells were infected with the guide RNA-Cas9 plasmids, and two vinculin knockout clones were obtained (KO vin cl.1 and KO vin cl.2). Vinculin expression was examined by immunoblotting whole cell lysates with an antibody against vinculin or β-actin as a loading control. The graph beneath the immunoblots depicts vinculin levels normalized to vinculin levels in the parental cells; the data are mean±s.e.m., *n*=3 biologically independent samples. (B) The vinculin-knockout cells were smaller and more rounded when compared to 4T1 parental cells. Representative phase contrast images of the cells are shown from *n*=3 experimental repeats. Scale bars: 100 µm. (C) Immunofluorescence revealed a lack of detectable vinculin in knockout cells. Parental or vinculin knockout cells were fixed and stained with antibodies against phalloidin and vinculin; actin and vinculin were examined by confocal microscopy. Representative images from *n*=3 experimental repeats are shown. Scale bars: 10 µm. (D) Protein expression is decreased in vinculin-knockout cells. Talin, E-cadherin and paxillin expression was examined by immunoblotting whole-cell lysates; β-actin was used as a loading control. The graphs beneath the blots show the amount of each protein normalized to actin. The data are mean±s.e.m., *n*=3 biologically independent samples. *P<0.05 (two-tailed unpaired Student's *t*-test).

To begin to identify the role of vinculin Y822 amino acid substitutions in cancer cells, vinculin was deleted using CRISPR/Cas9 from 4T1 mouse metastatic breast cancer cells. For this purpose, two guide RNAs were created and used to target the 5′ and 3′ ends of the vinculin gene. Using this approach, a 95±1% (mean±s.e.m.) reduction in vinculin expression was achieved (Fig. 1A). An initial examination of the cell morphologies using phase contrast imaging revealed that the knockout cells were more rounded compared to parental cells and they were unable to form close contacts with neighboring cells (Fig. 1B). To probe these phenotypes in more detail, the cells were stained for phalloidin and vinculin. In the parental 4T1 cells, vinculin was ubiquitously expressed throughout the cells and was enriched in focal adhesions (Fig. 1C). In contrast, vinculin was undetectable in the knockout cells, and the cells were more rounded than the controls (Fig. 1C). Finally, we probed for the levels of cell–cell junction and focal adhesion proteins in the parental and knockout cells, given that previous studies have indicated diminished levels of expression for these proteins in cells lacking vinculin (Subauste et al., 2005; Wang et al., 2019; Watabe-Uchida et al., 1998). Expression levels of paxillin, talin and E-cadherin proteins located at focal adhesions or cell–cell junctions, were significantly decreased (Fig. 1D). The decreased protein levels and cell morphologies of the vinculin knockout cell line generated in this study are in good agreement with the phenotypes previously reported when vinculin is deleted, thereby suggesting that our vinculin knockout system in cancer cells is behaving appropriately (Coll et al., 1995; Subauste et al., 2005; Volberg et al., 1995; Wang et al., 2019; Watabe-Uchida et al., 1998).

### Y822C and Y822F vinculin-expressing cells have different morphologies

**Fig. 2.**
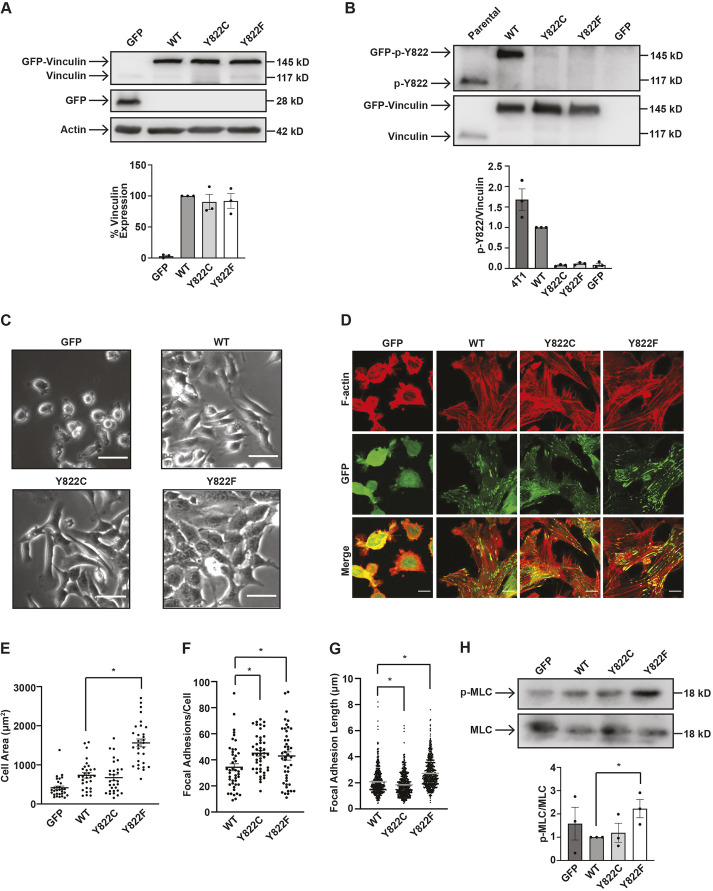
**Y822C and Y822F vinculin expressing cells have different focal adhesion morphologies.** (A) 4T1 vinculin-knockout cells were rescued with GFP-tagged versions of wild-type (WT) vinculin, Y822C or Y822F vinculin, or a GFP-only control. The expression of vinculin and GFP was examined by immunoblotting whole-cell lysates with an antibody against vinculin, GFP or β-actin as a loading control. The graph shows vinculin expression relative to total vinculin levels in the wild-type cells; data are mean±s.e.m., *n*=3 biologically independent samples. (B) The phosphorylation state of Y822 was examined in the cells. Lysates from the indicated cell lines were immunoblotted with antibodies against phosphorylated Y822 (p-Y822) then stripped and re-probed with antibodies that recognize total vinculin levels. The graph shows p-Y822 levels relative to total vinculin levels; data are mean±s.e.m., *n*=3 biologically independent samples. (C) The morphologies of the Y822C- and Y822F-expressing cells are altered. Representative phase contrast images of indicated cells from *n*=3 experimental repeats. Scale bars: 50 µm. (D–G) The focal adhesion phenotypes of Y822C and Y822F cells are different. (D) The indicated cells were fixed and stained with antibodies against phalloidin, and phalloidin and GFP expression was examined by confocal microscopy. Representative images are shown. Scale bars: 10 µm. (E–G) Cell area and focal adhesions containing GFP–vinculin were measured in ImageJ. (E) The cell area in the Y822F cells was larger compared to wild-type, Y822C and GFP-only cells. The graph shows total cell area, where the area of 30 cells was measured. *n*=3 biologically independent samples. (F) Y822C and Y822F vinculin-expressing cells showed an increased number of focal adhesions per cell compared to wild-type vinculin-expressing cells. The graph shows focal adhesion number per cell where focal adhesions in 45 cells were counted. *n*=3 biologically independent samples. (G) The focal adhesions in the Y822F cells were longer compared to wild-type cells, whereas the focal adhesions were smaller in the Y822C cells. The graph shows focal adhesion length where 665 focal adhesions were measured in 28 cells. *n*=4 biologically independent samples. Error bars in E–G are mean±s.e.m. (H) Myosin light chain phosphorylation (p-MLC) was elevated in Y822F cells. The cells were lysed and immunoblotted with antibodies that recognize phosphorylated MLC and then stripped and re-probed with antibodies that recognize total MLC levels. Results are mean±s.e.m.; *n*=3 biologically independent samples. *P<0.05 (two-tailed unpaired Student's *t*-test).

To investigate how Y822 mutations affect cancer cell function, vinculins harboring amino acid substitutions at residue Y822 were re-expressed into the knockout cells. For this, we expressed GFP-tagged versions of vinculin Y822F, a mutant protein unable to be phosphorylated, and vinculin Y822C, a cancer mutation identified in The Cancer Genome Atlas (TCGA) database. Cells re-expressing wild-type vinculin or GFP were included as controls. Immunoblotting of cell lysates from these cell lines revealed similar expression of the GFP-tagged proteins (Fig. 2A). To confirm that the cells containing Y822C and Y822F vinculins were unable to be phosphorylated at the Y822 residue on vinculin, phosphorylation of vinculin was examined. Compared to 4T1 parental cells and knockout cells re-expressing wild-type vinculin, cells containing Y822C and Y822F vinculin had significantly lower phosphorylation at Y822, with similar levels to that of the GFP control (Fig. 2B). An examination of the cells using phase-contrast imaging revealed that the wild-type, Y822C and Y822F cells appeared more spread and elongated than the GFP-only expressing cells (Fig. 2C). Additionally, the Y822F vinculin cells appeared flatter and seemed to pack in more closely with neighboring cells than the wild-type or the Y822C cells when plated at a lower density (Fig. 2C). When plated at a higher cell density, each cell line formed a compact monolayer (Fig. S1A) but did not form cell–cell adhesions, as evident from the lack of β-catenin staining in the GFP- or wild-type-expressing cells (Fig. S1B).

To examine the morphology in more detail, the cell lines were subjected to immunofluorescence. Phalloidin staining revealed that there were more robust actin stress fibers and more spreading in cells expressing wild-type, Y822C or Y822F vinculin when compared to the GFP controls (Fig. 2D). In many instances, it appeared that the cells expressing Y822F were more spread than cells expressing either wild type or Y822C vinculin. To determine whether the enhancement of cell spreading was significant, we measured the area of 30 cells from three independent experiments. This analysis revealed that the Y822F cells were significantly more spread than their wild-type or Y822C counterparts (Fig. 2E). Furthermore, a quantification of the number and length of focal adhesions revealed the Y822F-expressing cells might be more spread because they have more (Fig. 2F) and longer (Fig. 2G) focal adhesions. Interestingly, the Y822C cells had more and smaller focal adhesions (Fig. 2F,G).

Because we observed more cell spreading and larger focal adhesions in the Y822F cells, we determined whether the cells were more contractile. To test this possibility, we compared myosin light chain (MLC) phosphorylation in the various cells. In comparison to wild-type cells, myosin light chain phosphorylation was elevated in the Y822F cells (Fig. 2H). Thus, the Y822F-expressing cells are more spread, have larger focal adhesions, and are more contractile than cells expressing Y822C or wild-type vinculin, thereby suggesting that the two Y822 mutations differentially affect focal adhesion biology in cancer cells.

### Y822C and Y822F vinculin have different effects on cell biology

**Fig. 3.**
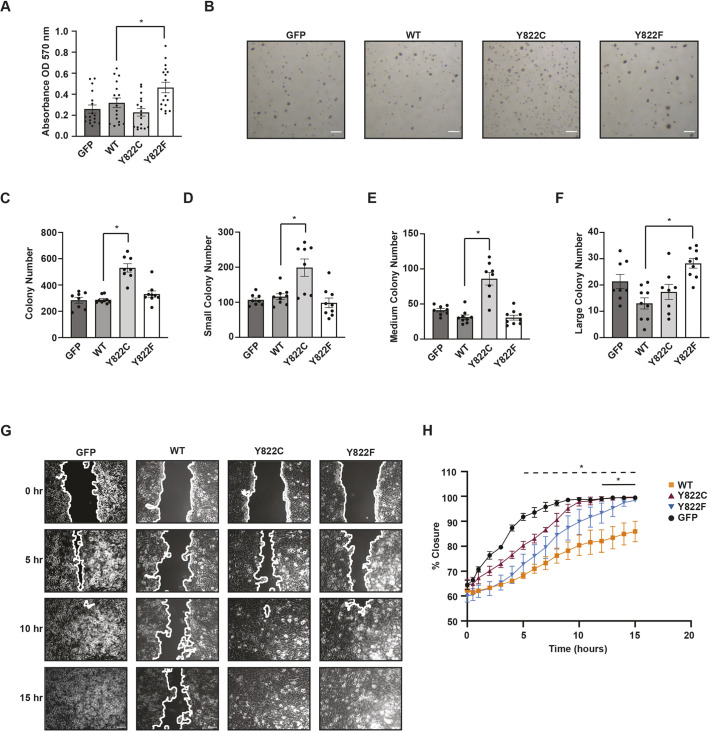
**Y822C and Y822F vinculin-expressing cells have increased tumorigenicity.** (A) 4T1 Y822F vinculin-expressing cells proliferated more than wild-type (WT) vinculin-expressing cells. Cells expressing the indicated GFP proteins were seeded into 96-well plates. After 72 h, cell viability was determined by means of an MTT assay. Results are mean±s.e.m.; *n*=5 biologically independent samples. (B–F) Y822C and Y822F cells proliferated more than wild-type vinculin cells in soft agar. (B) The indicated cells were seeded in 0.35% agarose and plated over a solidified 0.5% agarose bottom layer. The cells were incubated for 4 weeks. Representative images are shown; *n*=3 biologically independent samples. Scale bars: 1 mm. (C) Y822C cells grew more colonies than wild-type cells. Colony number was counted in ImageJ. *n*=3 biologically independent samples. (D–F) Y822C and Y822F colony size was different compared to wild-type cells. Colony size was counted with the Multi-point tool in ImageJ using three differently sized circles corresponding with small, medium and large sized colonies. (D,E) Y822C cells grew more small and medium colonies, respectively, whereas Y822F vinculin expressing cells grew more large colonies compared to wildtype cells (F). Results are mean±s.e.m.; *n*=3 biologically independent experiments. *P<0.05 (two-tailed unpaired Student's *t*-test). (G) representative images from wound healing assay for indicated cells; the edges of the wound are marked. Y822C and Y822F cells migrated faster than wild-type cells. Scale bars: 100 μm. (H) Y822F cells migrated to close a wound at a significantly faster rate than wild-type vinculin cells starting after 12 h of migration (significance denoted by solid black line). Y822C cells migrated to close the wound significantly faster than wild-type vinculin cells starting after 5 h of migration (significance denoted by dashed black line). Results are mean±s.e.m.; *n*=3 biologically independent experiments. *P<0.05 (two-way ANOVA with post-hoc Tukey's multiple comparison test).

To determine whether the differences in spreading and focal adhesion morphology in the Y822C and Y822F altered the biological response of the cells, we assessed their growth and migration. As a first measure of cell proliferation, we plated the GFP, wild-type, Y822C or Y822F cells and monitored the number of live cells after 72 h using a 3-(4,5-dimethylthiazol-2-yl)-2,5-diphenyl-2H-tetrazolium bromide (MTT) assay. The Y822F vinculin cells proliferated to a significantly higher extent than wild-type cells, whereas Y822C vinculin cells proliferated similarly to wild-type or GFP-expressing cells (Fig. 3A). As a second measure of proliferation, we monitored growth in soft agar. After 4 weeks, colony number and size were examined for each cell line. Compared to wild-type cells, the total number of colonies and both the number of small and medium colonies were significantly increased in Y822C cells (Fig. 3B–E). The different behaviors of the Y822C vinculin in the MTT and soft agar assays likely reflects an ability to proliferate differently in anchorage-dependent versus anchorage-independent environments. In contrast to Y822C cells, the number of total, small and medium colonies in the Y822F vinculin cells were comparable to the number for wild-type cells (Fig. 3B–E). However, the Y822F vinculin cells showed a significant increase in large colony number compared to wild-type cells, whereas the Y822C vinculin cells showed similar levels (Fig. 3F). Thus, the Y822 mutant vinculins behave differently from the wild-type protein in soft agar assays.

To test whether cell migration was affected by the differences in focal adhesion morphology between Y822C- and Y822F-expressing cells, confluent monolayers were scratched, and the ability of the cells to close the wound was monitored. Consistent with previous studies, cells expressing wild-type vinculin closed the wound more slowly than cells lacking vinculin (Lee et al., 2019b; Saunders et al., 2006; Subauste et al., 2004; Xu et al., 1998). The Y822C- and Y822F-expressing cells closed the wound more quickly than cells expressing wild-type vinculin. However, the initial rate of closure was different; both Y822C- and Y822F-expressing cells began closing the wound significantly earlier than wild-type, but starting at 5 hours for Y822C and 12 h for Y822F (Fig. 3G,H). These results suggest that Y822C cells initially migrate faster than Y822F and wild-type cells.

### Y822C and Y822F vinculin mutants recruit ligands differently

**Fig. 4.**
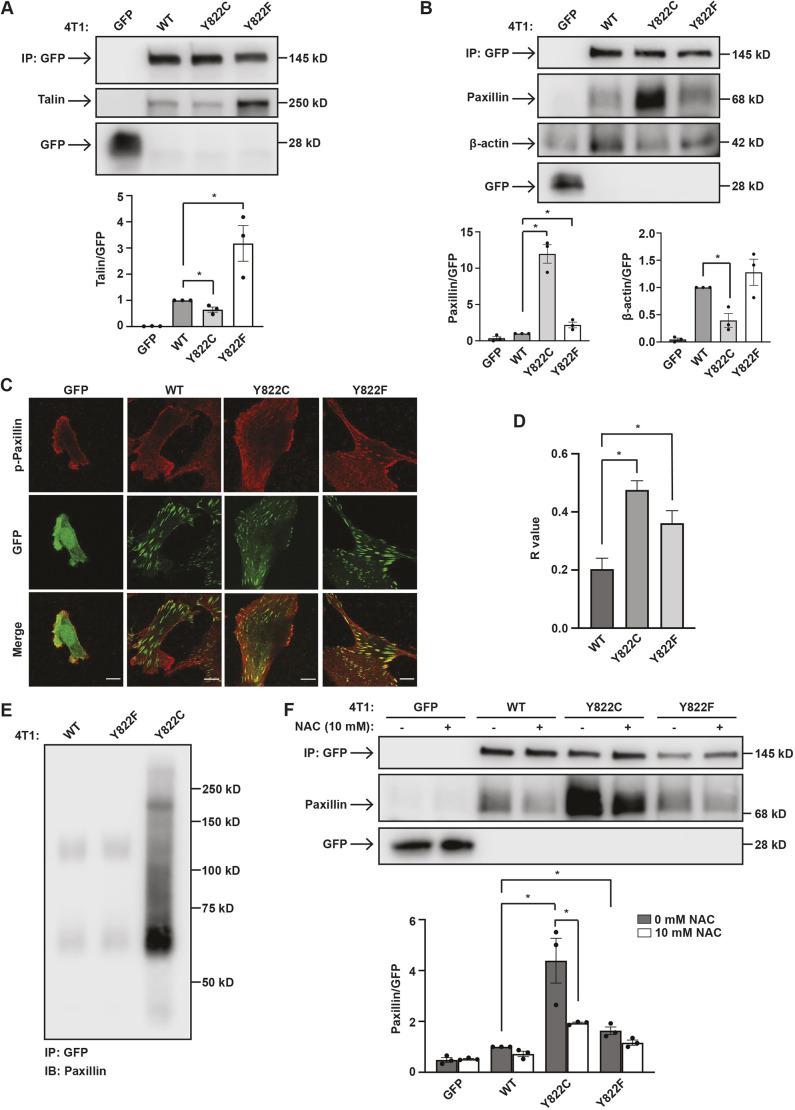
**Y822C and Y822F vinculin mutants bind differently to binding partners and the cysteine at Y822C vinculin is necessary for this effect.** (A) Y822F bound talin better than wild-type (WT) vinculin; Y822C bound talin less well. GFP or the GFP-tagged vinculins were immunoprecipitated, and the co-precipitating levels of talin were examined (IP). Average talin binding is shown relative to GFP or GFP–vinculin levels. Data are mean±s.e.m., *n*=3 biologically independent samples. (B) Y822C vinculin co-precipitated with more paxillin but less β-actin compared to wild-type vinculin. Y822F vinculin co-precipitated with more paxillin, but similar amounts of β-actin compared to wild-type vinculin. GFP or GFP-tagged vinculin was immunoprecipitated from 4T1 cells, and the co-precipitating levels of paxillin and β-actin were examined (IP). Average paxillin or β-actin binding is shown relative to GFP or GFP-tagged vinculin levels. Data are mean±s.e.m., *n*=3 biologically independent samples. (C,D) More phosphorylated paxillin colocalized with Y822C vinculin. (C) The indicated cell lines were stained with antibodies against phosphorylated paxillin (p-Paxillin) and visualized using confocal microscopy. Representative images are shown. Scale bars: 10 µm. (D) The graphs depict a R value for the colocalization of phospho-paxillin and vinculin. The analysis revealed more phosphorylated paxillin colocalized with Y822C vinculin-containing focal adhesions. Cells from three separate field of views were chosen. Data are mean±s.e.m., *n*=3 biologically independent samples. *P<0.05 (ordinary one-way ANOVA and Dunnett's multiple comparisons tests). (E) The association of vinculin mutants with paxillin under non-reducing conditions. GFP-tagged vinculins were immunoprecipitated (IP:GFP) from cells, run on a gel under non-reducing conditions, and then probed for the presence of paxillin or GFP-vinculin (IB: Paxillin). The graph depicts the amount of paxillin recovered as a function of GFP–vinculin levels. Data are mean±s.e.m., *n*=3 biologically independent samples. (F) Pre-incubation of cells with the antioxidant NAC decreased paxillin binding to Y822C vinculin in 4T1 cells and does not significantly change paxillin co-precipitation with wild-type or Y822F vinculin. Cells were pre-treated with 10 mM NAC for 1 h then GFP or GFP–vinculin was immunoprecipitated, and the co-precipitating levels of paxillin were examined. Average paxillin binding is shown relative to GFP or GFP–vinculin levels. Data are mean±s.e.m., *n*=3 biologically independent samples. *P<0.05 (two-tailed unpaired Student's *t*-test).

Given Y822C and Y822F affected focal adhesion number and length differently (Fig. 2D,F,G), we tested whether the substitutions at Y822 might alter recruitment of binding partners. To test this possibility, the GFP proteins were immunoprecipitated and association of head and tail ligands was examined. There was no detectable recruitment of talin, a vinculin head ligand, to GFP alone. In contrast, wild-type vinculin recruited talin (Fig. 4A) as previously described (Burridge and Mangeat, 1984). We consistently observed a small, but statistically significant, decrease in talin recruitment to vinculin in the Y822C cells when compared to wild-type-expressing cells. Surprisingly, the amount of talin recruited to Y822F vinculin was robustly increased (Fig. 4A). Increased talin recruitment has previously been observed to a mutant vinculin known as T12, which harbors several amino acid substitutions that relieve head–tail autoinhibition (Cohen et al., 2005). To determine whether the increased talin recruitment to vinculin Y822F was akin to what is observed in cells expressing the T12 vinculin variant, we compared the level of talin recruitment by co-immumoprecipitation. In these studies, Y822F vinculin bound talin to a significantly greater extent than did wild-type vinculin, but this level was ∼390-fold less than the amount of recovered with T12 (Fig. S2A). Additionally, we replaced Y822 with a glutamine residue to maintain any potential hydrogen bonding with neighboring residues and maintain the hydrophilic character of the tyrosine residue. We observed that Y822Q vinculin bound talin significantly better than the wild-type but to the same extent as Y822F (Fig. S2A). Taken together, these data suggest the Y822F binds talin to a different extent from mutant vinculin proteins, such as T12, which are constitutively in the open conformation.

To determine whether the recruitment of other binding partners was altered by substitution at Y822, we assessed binding of paxillin and β-actin – two vinculin tail ligands. In these studies, β-actin and paxillin bound wild-type vinculin as previously described (Huttelmaier et al., 1997; Johnson and Craig, 1995b; Turner et al., 1990) (Fig. 4B). We observed small, yet statistically significant, increases in paxillin binding to Y822F as has previously been reported (Subauste et al., 2004) (Fig. 4B). β-actin co-immunoprecipitation was unaffected by the Y822F mutation. Strikingly, paxillin co-immunoprecipitated with Y822C vinculin to a significantly higher extent than wild-type vinculin, but it co-immunoprecipitated with β-actin to a significantly lower extent (Fig. 4B). To ensure that the altered ligand recruitment was not a consequence of altered protein expression, we examined expression of the focal adhesion components lost in vinculin-knockout cells (Fig. 1D). This analysis revealed that the expression of talin and paxillin were similar in the wild-type-, Y822C- and Y822F-expressing cell lines, suggesting that the increased paxillin or talin binding were not the result of changes in their expression (Fig. S2B).

To determine whether these results were specific to cancer cells, the same vinculin amino acid substitutions at Y822 were introduced into MCF-10A mammary epithelial cells in which endogenous vinculin levels were suppressed using shRNAs. Binding of the GFP-tagged vinculins to paxillin was examined. In MCF-10A cells expressing Y822C vinculin, paxillin co-immunoprecipitated to a significantly higher extent compared to wild-type cells, whereas in Y822F cells, a modest yet significant increase in paxillin co-immunoprecipitation with Y822F vinculin compared to wild-type cells was observed (Fig. S2C). Taken together, these results indicate that the substitution at Y822 differentially affects ligand binding irrespective of cell type.

Previous studies indicate that paxillin phosphorylation promotes its binding to vinculin (Pasapera et al., 2010). Thus, we examined whether Y822C vinculin colocalized with the phosphorylated form of paxillin. We observed significantly more phosphorylated paxillin co-localizing with Y822C and to a lesser extent Y822F vinculin than with wild-type vinculin (Fig. 4C,D). This observation could be accounted for by a significant elevation in phosphorylated paxillin in the Y822C and Y822F cells (Fig. S2D).

Given that cysteine residues contain a thiol side chain, which is susceptible to oxidation to form disulfide bonds with other thiol-containing molecules, we considered that the cysteine residue at 822 on vinculin could form a disulfide bond to increase binding with paxillin. To test this possibility directly, we examined whether Y822C was forming a disulfide with paxillin by examining association under non-reducing conditions. For this, we immunoprecipitated GFP-tagged vinculins from cells, resuspended the immunopreciptates in sample buffer without β-mercaptoethanol, separated the proteins on an SDS-PAGE gel and immunoblotted the immunoprecipitates with antibodies against paxillin. We observed that Y822C vinculin, but not Y822F or wild-type protein bound paxillin in this non-reducing environment as evident by the presence of a band that migrated at ∼184 kDa, which is the predicted molecular mass of paxillin–vinculin dimers (Fig. 4E). We also noted the presence of a monomeric paxillin band, which likely reflects that the protein was partially reduced in the lysis buffer. To confirm the presence of a disulfide bond using another approach, co-immunoprecipitation of paxillin with the GFP-tagged vinculins was examined in cells expressing the mutant vinculins that were treated with 10 mM NAC – a prominent breaker of disulfide bonds. In wild-type- and Y822F-expressing cells, the addition of NAC did not significantly change the amount of paxillin that co-immunoprecipitated with GFP–vinculin (Fig. 4F). In contrast, the addition of NAC significantly reduced paxillin recruitment to GFP–vinculin Y822C (Fig. 4F), suggesting the critical importance of disulfide bond formation.

**Fig. 5.**
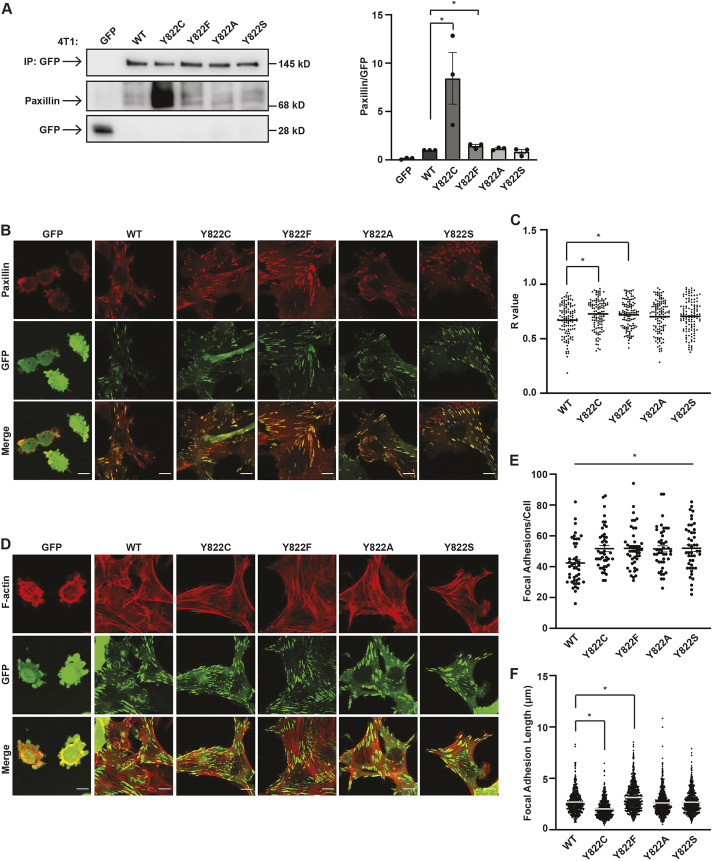
**A serine or alanine substitution at Y822 does not affect paxillin binding or focal adhesion length.** 4T1 cells stably expressing the indicated vinculin variants were generated. (A–C) The Y822A or Y822S mutations abrogated the effect of Y822C on paxillin binding and co-localization with vinculin. WT, wild-type. (A) GFP or GFP-tagged vinculin was immunoprecipitated, and the co-precipitating levels of paxillin were examined. Average paxillin binding is shown relative to GFP or GFP-vinculin levels. Data are mean±s.e.m., *n*=3 biologically independent samples. (B,C) Cells were stained with antibodies against paxillin and the colocalization of paxillin with the GFP proteins was examined. Representative images are shown (B). Scale bars: 10 µm. The graph in C represents a quantification of the amount of paxillin as a function of vinculin in the focal adhesions revealed more paxillin colocalized with Y822C- and Y822F-vinculin-containing focal adhesions. Graphs indicating intensity show fluorescence intensity of the ROI of paxillin within the focal adhesion. Data are mean for *n*=3 biologically independent samples. *P<0.05 (ordinary one-way ANOVA and Dunnett's multiple comparisons tests). (D–F) Y822A and Y822S cells had focal adhesion phenotypes similar to those in wild-type cells. (D) GFP or GFP-tagged vinculin-expressing cells were fixed and stained with antibodies against phalloidin; GFP expression was examined by confocal microscopy. Representative images are shown. Scale bars: 10 µm. (E) Y822C, Y822F, Y822A and Y822S vinculin cells showed increased number of focal adhesions per cell compared to cells expressing wild-type vinculin. Focal adhesions containing GFP–vinculin were counted in ImageJ. Graph shows focal adhesion number per cell where focal adhesions in 45 cells were counted. Data are mean±s.e.m., *n*=3 biologically independent samples. (F) Y822A and Y822S vinculin cells had similarly sized focal adhesions to those in wild-type cells. Focal adhesions containing GFP–vinculin were measured in ImageJ. The graph shows focal adhesion length where 666 focal adhesions were measured in 27 cells. *n*=3 biologically independent samples. *P<0.05 (two-tailed unpaired Student's *t*-test).

To explore the requirement for disulfide bond formation, we determined whether the increased binding of Y822C to paxillin required a cysteine residue with a side chain sulfhydryl group or whether other amino acids substitutions would sustain increased recruitment. Vinculin with tyrosine to serine or alanine replacements at Y822 were generated and expressed in the vinculin-knockdown cells. A serine replacement was chosen because this residue is a similar size to a cysteine residue but contains a hydroxyl group rather than a sulfhydryl group. An alanine replacement was chosen because of its similar size to cysteine and its lack of a sulfhydryl group. GFP-tagged versions of Y822A or Y822S vinculin were expressed to similar levels to the wild-type protein in the vinculin-knockout cell lines (Fig. S2E). Co-immunoprecipitation (Fig. 5A) and colocalization (Fig. 5B,C) studies revealed that replacement of Y822C with Y822A or Y822S reduced paxillin recruitment to vinculin to wild-type levels, suggesting that a cysteine residue is critical for increased paxillin recruitment.

To determine whether the focal adhesion phenotypes observed with Y822C vinculin were negated by expression of Y822A or Y822S vinculin, the cells were examined using immunofluorescence (Fig. 5D). Like Y822C, the Y822A and Y822S cells had more focal adhesions than wild-type cells (Figs 5D,E, 2E). In contrast, the size of focal adhesions in the Y822A or Y822S cells were comparable to those in wild-type, and not Y822C, cells (Figs 5D,F, 2F). Taken together, these data suggest that the increased recruitment of paxillin to vinculin modulates focal adhesion size.

## DISCUSSION

Many studies have focused on how loss or overexpression of mechanotransduction pathway components contribute to cancer progression. However, the effect of amino acid substitutions that specifically interfere with mechanotransduction are less well understood, especially in the context of cancer. Here, we sought to determine, in a cancer model, the consequences of perturbing the mechanosensitive protein vinculin at Y822, an amino acid residue phosphorylated in response to force. The Y822C change, a mutation found in some human cancers, produced cells with numerous, small focal adhesions that grew better in soft agar than control cells. We determined the mechanism changes and provide evidence that Y822C vinculin binds phosphorylated paxillin, a vinculin tail ligand, more robustly. Moreover, we show the mechanism for the increased binding is a disulfide bond formation between vinculin and paxillin. Although mutation of Y822C affects paxillin binding, it was notable that mutation of Y822F increased talin binding, thereby producing larger focal adhesions and more contractility in the cell (Figs 4A, 2D–H). Taken together, these data suggest that vinculin Y822 modulates the recruitment of vinculin binding partners.

How substitution of different amino acids at Y822 affects ligand binding is intriguing. The known binding sites for paxillin and talin are in different regions of the protein and both are far from Y822 (Fig. S2F). Paxillin binds to a region of 50 amino acids near the vinculin C-terminus spanning residues 979–1028 (Wood et al., 1994). In contrast, talin binds to the vinculin head domain D1 (Bois et al., 2006; Lee et al., 2008; Roberts and Critchley, 2009; Yao et al., 2014). Both are far away from the D4 domain of vinculin where Y822 lies. One possibility is that mutation of Y822 induces global structural changes in vinculin that alter binding. Our data do not support this notion as all the vinculin Y822 mutant proteins described in this study localize to focal adhesions and rescue cell migration and proliferation defects of the vinculin-knockout cells (Figs 2D, 3 and 5D). Another possibility is that introduction of amino acid substitutions at Y822 do not completely unfold the protein but rather produce conformational changes that promote ligand binding. It is well established that vinculin exists in at least two conformations, a closed, autoinhibited conformation and an open conformation. The D1 domain in the vinculin head interacts with the D5 vinculin tail domain to maintain autoinhibited vinculin (Bakolitsa et al., 2004; Borgon et al., 2004; Cohen et al., 2005; Izard et al., 2004; Izard and Vonrhein, 2004). However, later work, which utilized structural and biochemical techniques defined a second autoinhibitory site between residues 710–836 and the vinculin tail (Cohen et al., 2005). Consistent with this notion, more recently, vinculin has been reported to exist in semi-open conformational states (Chorev et al., 2018). One of those predicted states has the D4 domain unfolding as a first step in promoting conformational changes leading to vinculin activation (Garakani et al., 2017). Because the 822 residue is within the D4 domain, it is possible that mutation could be promoting partially activating conformational changes. Such conformational changes could increase the probability of a binding site on vinculin being exposed to allow for protein binding.

The increased paxillin recruitment to Y822C vinculin is striking. Increased recruitment requires a cysteine residue substitution. Preventing phosphorylation using a Y822F mutation only had a modest effect on paxillin binding (Figs 4B and 5A; Fig. S2C). Moreover, the increased paxillin recruitment did not appear to result from introducing a bulky residue as replacement with a simple amino acid, alanine, at Y822 had no effect on paxillin binding (Fig. 5A–C). Our observation that substitution of a serine at Y822 also had no effect on paxillin binding (Fig. 5A–C) suggests a critical importance of the sulfhydryl group for increased paxillin recruitment. Indeed, Y822C and paxillin co-associated under non-reducing conditions (Fig. 4E) and treatment of cells with NAC, a thiol compound that possesses a free sulfhydryl group through which it reduces disulfide bonds, reduced paxillin binding to near wild-type levels (Fig. 4F).

How the addition of a sulfhydryl group promotes increased paxillin recruitment is of interest. The most likely possibility is that the sulfhydryl group allows paxillin–vinculin dimers to form. When we immunoprecipitated vinculin Y822C from lysates and resuspended the immunoprecipitates in sample buffer without β-mercapoethanol and separated then on a reducing gel, a band that corresponded to the molecular mass of vinculin–paxillin dimers was detected (Fig. 4E). Of note, the level of vinculin–paxillin dimers could be greater than the blots reveal as cysteine-crosslinked complexes are much hard to elute from beads under non-reducing than reducing conditions. Another distinct possibility is that vinculin itself dimerizes, as has previously been reported (Abé et al., 2011; Chinthalapudi et al., 2015; Huttelmaier et al., 1997; Janssen et al., 2006; Johnson and Craig, 2000), and the vinculin dimer binds paxillin better. Alternatively, Y822 could form an intramolecular disulfide with nearby cysteine residues, which might stabilize the vinculin tail or relieve the vinculin head–tail interaction to promote paxillin binding. Our data argue against a role for the intramolecular disulfide bond. We do not observe multiple vinculin bands when we examine Y822C under non-reducing conditions (Fig. 4E). Additionally, the binding of Y822C is specific to paxillin, and not to other vinculin ligands, many of which have cysteine residues. Finally, Y822C vinculin binds talin slightly less than wild-type, arguing against head–tail severing. Therefore, it is unlikely that Y822C is forming an intramolecular disulfide bond that affects paxillin binding. More work is needed to determine the precise mechanism of binding.

This study also provides insight into how mutation of Y822C might confer an advantage to cancer cells. Previously, we reported that Y822 was localized preferentially in cell–cell adhesions and was tyrosine phosphorylated in response to mechanical force. The mutation of Y822C might prevent phosphorylation, thereby allow phenotypic switching of the vinculin in cell–cell adhesions to focal adhesions, thereby reducing cell–cell adhesion. In focal adhesions, Y822C binds paxillin. Enhanced paxillin recruitment can alter focal adhesion shape. Following integrin engagement, vinculin and paxillin are some of the first proteins recruited to cell–matrix adhesions (Ballestrem et al., 2006; Zaidel-Bar et al., 2003; Zimerman et al., 2004), and the interaction is critical for formation of small nascent adhesions (Burridge et al., 1992). Expression of Y822C vinculin produces cells with numerous, small adhesions. These nascent adhesions generate strong propulsive forces at the leading edge of migrating cells (Beningo et al., 2001), providing one possible explanation for the increased migratory capacity observed in cells expressing Y822C. Additionally, the paxillin that is recruited to Y822C vinculin is tyrosine phosphorylated, and tyrosine-phosphorylated paxillin binds numerous effectors linked to increased proliferation. In this way, Y822C vinculin-expressing cancer cells have increased migratory and proliferative capacities – two attributes essential for tumors to grow and metastasize to distant sites.

In summary, this work demonstrates that Y822 vinculin modulates ligand binding, focal adhesion morphology, proliferation and migration in cancer cells. We show that replacing the tyrosine with a cysteine at residue 822 on vinculin causes it to behave differently from changing it to a phenylalanine, suggesting that there are benefits to distinct mutations in disease states. This information provides insight into how mutations that disrupt mechanotransduction in cancer can differentially affect cell function to promote disease progression. It also provides new insight into how vinculin conformations are modulated and control focal adhesion dynamics and biological outcomes.

## MATERIALS AND METHODS

### Cell lines

4T1 mouse mammary cancer cells containing luciferase were a generous gift from Dr Christopher Stipp of the University of Iowa (Zhou et al., 2014) and MCF-10A human breast epithelial cells were purchased from ATCC and were maintained as previously described (Bays et al., 2017, 2014). All cell lines and their derivatives were used for no more than 8 passages and were periodically checked for mycoplasma contamination. All 4T1 cells were maintained in medium with G418 (0.8 mg ml^−1^; Research Products International; #G64000). 4T1 vinculin-knockout cells were made with the help of Genscript (Piscataway, NJ). 4T1 cells expressing GFP, GFP–wild-type vinculin, GFP–Y822C vinculin, GFP–Y822F vinculin, GFP–Y822A vinculin, GFP–Y822S vinculin, GFP–Y822Q vinculin or GFP–T12 vinculin were selected for GFP via flow cytometry and maintained in medium with G418 (0.8 mg ml^−1^). MCF-10A vinculin knockdown cells were made as previously described (Bays et al., 2017) and were selected and maintained in puromycin (2 μg ml^−1^). MCF-10A vinculin knockdown cells expressing GFP, GFP–wildtype vinculin, GFP–Y822C vinculin or GFP–Y822F vinculin were selected and maintained in medium with puromycin (2 μg ml^−1^; Research Product International; #P33020) and G418 (0.8 mg ml^−1^). The 293 GPG cells are a virus-producing cell line that are a derivative of 293T cells. The 293 GPG cells were maintained as previously described (Bays et al., 2014).

### Constructs

To inhibit expression of proteins using CRISPR, four distinct guide RNA sequences targeting the 5′ and 3′ end of the vinculin gene were inserted into pSpCas9 BB-2A-GFP PX458 (Genescript). The sequences targeted were: 5′-TGTGGGGCTAGTTACGCCGAGGG-3′, 3′-CATCTCTAACACGTCATTCAGGG-5′, 5′-CTTCAGAATCTGATCCTCAGTGG-3′ and 3′-AGGTCTTGAGAGCCTATAGTGGG-5′.

cDNAs containing GFP-tagged wild-type, Y822C, Y822F, Y822A, Y822S, Y822Q or T12 vinculin were made as previously described (Bays et al., 2014). Briefly, GFP-tagged Y822C, Y822F, Y822A, Y822S, Y822Q, or T12 vinculin mutants were prepared using site-directed mutagenesis to introduce the respective amino acid substitutions into a pLEGFP-C1 plasmid containing full-length wild-type chicken vinculin (Bays et al., 2014; Campbell et al., 2018).

### Virus production and infection

Retroviruses were produced as previously described (Bays et al., 2014). Briefly, 293 GPG cells were transfected, and viruses were produced. Before infection, 4T1 cells were grown to 60% confluency. On the day of infection, cells were washed twice with serum-free RPMI medium, incubated in OPTI-MEM (Gibco BRL, Gaithersburg, MD) containing 4 μg ml^−1^ polybrene, and the viral particles were concentrated and incubated with the cells for 4 h at 37°C. Growth medium was then added to the cultures. The cells were then selected and sorted for GFP by using flow cytometry (Bays et al., 2014).

### Immunofluorescence images and quantification

Coverslips were placed in 24-well plates and coated with human fibronectin (10 μg ml^−1^) and cells were plated and grown at either low or high confluences. Cells were fixed with 4% paraformaldehyde for 10 min, permeabilized with 0.1–0.5% Triton X-100 for 3 min and washed with universal buffer (150 mM NaCl, 50 mM Tris-HCl, pH 7.6, and 0.01% NaN_3_) or phosphate-buffered saline (PBS). Cells were blocked with 5–10% BSA (Sigma-Aldrich, St. Louis, MO, USA) in universal buffer or PBS at 4°C overnight. Cells were incubated with primary antibody in 5–10% BSA in universal buffer or PBS for 1 h at room temperature, washed with PBS, incubated with secondary antibody in 5–10% BSA in universal buffer or PBS for 1 h at room temperature, washed with PBS and mounted with MOWIOL on a glass slide. Primary antibodies used were: vinculin monoclonal antibody at 1:200 (Sigma-Aldrich; V9131), paxillin conjugated with Alexa Fluor 647 at 1:200 (Abcam, Cambridge, MA, USA; ab246719), phospho-paxillin monoclonal antibody at 1:250 (Santa Cruz Biotechnology, Dallas, TX, USA; sc-365020), F-actin was stained using phalloidin conjugated with Alexa Fluor 594 at 1:500–1:1000 (Thermo Fisher Scientific, Waltham, MA, USA; A12379), and polyclonal β-catenin antibody at 1:500 (Sigma; C2206). Secondary antibodies used were: monoclonal goat anti-mouse-IgG conjugated with Alexa Fluor 488, monoclonal goat anti-rabbit-IgG conjugated with Alexa Fluor 594, and monoclonal goat anti-mouse-IgG conjugated with Alexa Fluor 647 antibody, all at 1:500–1000 in blocking buffer (5–10% BSA). Fluorescence images were captured at room temperature with a confocal microscope (model LSM 710; Carl Zeiss Micro Imaging). A 40× oil objective lens (Carl Zeiss Micro Imaging) with a numerical aperture of 1.3 was used. Images were obtained using the Zen2009 software (Carl Zeiss Micro Imaging). Quantifications of images were made using ImageJ software (Version 2.1.0/1.53c). Colocalization data was analyzed as follows. Image dimensionality was reduced. Focal adhesions were identified by using a 2.74% thresholding within the GFP–vinculin expression, making the image binary. Using the binary channel, focal adhesion regions of interest (ROIs) were captured using the wand (tracing) tool in ImageJ. The ROIs from the GFP–vinculin channel were then used to measure the paxillin or phosphorylated (p)-paxillin intensities. To measure colocalization of vinculin and paxillin or p-paxillin, the ImageJ base function (colocalization threshold) was used. Here, the ROI of a cell was measured between GFP–vinculin and the paxillin or p-paxillin channels. Colocalization values are a measure of the R_total_. Graphs indicating intensity show fluorescence intensity of the ROI of paxillin or p-paxillin within the focal adhesion. Data are represented by scatter plots.

### Phase images

Phase images of live cells were captured at room temperature with an inverted microscope (Axiovert 200M; Carl Zeiss), equipped with an ORCA-ERA 1394 HD camera (Hamamatsu Photonics, Hamamatsu City, Japan). A ×10 EC Plan Neofluor objective (NA 0.55; Carl Zeiss) was used for capturing phase images. Images were acquired using Axiovision 4.7 software (Carl Zeiss).

### Immunoprecipitation and western blotting

For immunoprecipitation experiments, cells were washed twice in cold PBS, lysed with cold EB lysis buffer (1 mM Tris-HCl, pH 7.6, 50 mM NaCl, 1% Triton X-100, 5 mM EDTA, 50 mM NaF, 20 μg ml^−1^ aprotinin, 2 mM Na_3_VO_4_ and 1 mM PMSF) or cold RIPA buffer (10 mM Tris-HCl, pH 8.0, 1 mM EDTA, 1% Triton X-100, 0.1% sodium deoxycholate, 0.1% SDS, 140 mM NaCl, 20 μg ml^−1^ aprotinin, 2 mM Na_3_VO_4_ and 1 mM PMSF). Cells lysed in EB buffer were centrifuged at 12,000 ***g*** and the supernatant was collected. Cells lysed in RIPA buffer were pre-cleared with protein A/G agarose beads (Thermo Fisher Scientific; 20421) and centrifuged at 12,000 ***g*** and the supernatant was collected. A 1:125 dilution of GFP antibody (Millipore Sigma; 11814460001) or a 1:125 dilution of vinculin antibody (Sigma-Aldrich; V9131) was incubated with the supernatant and complexes were recovered using protein A/G agarose beads, washed five times, resuspended in 2× sample buffer (200 mM Tris-HCl, pH 6.8, 20% glycerol, 5% β-mercaptoethanol, 4% SDS, 15 mg bromophenol blue, brought up to 50 ml with ddH_2_O), boiled for 10 min at 100°C, fractionated using SDS-PAGE and transferred to a PVDF membrane (Millipore Sigma; IPVH00010). For the non-reducing studies, cells were lysed in RIPA, vinculin was immunoprecipitated using G989, a polyclonal antibody raised against full-length chicken vinculin, which was a generous gift of Keith Burridge (University of North Carolina, NC, USA), and the resulting immunoprecipitates were washed and resuspended in 2× sample buffer without 2- mercaptoethanol, separated using SDS-PAGE and transferred to PVDF membrane. The membranes were blocked in 5% BSA in 1× tris-buffered saline with 0.1% Tween 20 (TBST) for talin, p-paxillin and p-MLC or 5% milk in TBST for vinculin, GFP, paxillin, MLC, β-actin and E-cadherin for 1 h at room temperature. The membranes were incubated with the following antibodies overnight at 4°C. Primary antibodies used for western blotting were: monoclonal GFP at 1:1000 (Millipore Sigma; 11814460001), monoclonal vinculin at 1:1000 (Sigma-Aldrich; V9131), monoclonal E-cadherin at 1:1000 (BD Biosciences, Franklin Lakes, NJ, USA; 610181), polyclonal pMLC at 1:1000 (Santa Cruz Biotechnology; 3674S), monoclonal β-actin at 1:1000 (Santa Cruz Biotechnology; 3700S), polyclonal MLC at 1:1000 (Santa Cruz Biotechnology; 3672S), monoclonal paxillin at 1:1000 (BD Biosciences; 610052), monoclonal p-paxillin at 1:1000 (Santa Cruz Biotechnology; sc-365020), polyclonal phosphoY822 vinculin (Abcam; ab200825), and monoclonal talin at 1:250 (Sigma-Aldrich; T3287). The membranes were washed three times in TBST and incubated with 1:1000 anti-mouse-IgG conjugated with horseradish peroxidase or anti-rabbit IgG conjugated with horseradish peroxidase (both Jackson Labs, Bar Harbor, MA, USA). The membranes were washed three times in TBST, visualized by using chemiluminescence detection reagents (Thermo Fisher Scientific; 34580), and the signal was detected on an Odyssey Fc Imager (Li-Cor Biosciences, Lincoln, NE, USA). For analysis, quantification of each assay was performed using Image Studio Lite (version 5.2.5) and quantification represents a minimum of three experiments, presented as mean±s.e.m. Ratios of phosphorylated proteins to total proteins were quantified by stripping and re-probing membranes. Statistical analysis was conducted using two-tailed unpaired Student's *t*-tests. Uncropped images of western blots from this study are shown in Figs S3, S4.

### Soft agar assay

The base layer of 0.5% agarose containing medium was plated into six-well plates (2 ml/well) and allowed to solidify at room temperature for 1 h. 10^4^ cells/2 ml in 0.35% agarose was added to form the top layer and was allowed to solidify at room temperature for 3 h. A feeder layer of medium was added after 1 day and then added weekly until completion of the assay. Colonies were allowed to grow for 4 weeks at 37°C and 5% CO_2_ before imaging. Soft agar images were acquired using a Leica S6D microscope, equipped with a Leica MC120 HD camera (Leica Microsystems, Wetzlar, Germany). Colonies were quantified using ImageJ to count colony number and size. Each experiment had two or three technical replicates and was repeated at least three times. Statistical analysis was conducted using two-tailed unpaired Student's *t*-tests.

### Wound healing studies

4T1 cells were plated on 35 mm plates and allowed to grow until they formed a monolayer. A wound was created in the monolayer by using a pipette tip. The cells were then imaged using an inverted light microscope (Axiovert 200M; Carl Zeiss) and imaged using an ORCA-ER camera (Hamamatsu Photonics, Hamamatsu City, Japan) using Axiovision software (Carl Zeiss) while in a 37°C chamber at 5% CO_2_ humidity. The wound closure was captured over 15 h with the imaging software taking a picture every 5 min. ImageJ software was used to quantify the area of the wound over time. An ImageJ Plugin titled Wound_healing_size_tool.ijm designed by Juan Cruz's group (Suarez-Arnedo et al., 2020) was utilized to manually quantify images. Wound healing experiments were repeated at least three times. Statistical analysis was conducted using a two-way ANOVA with post-hoc Tukey's multiple comparison test.

### MTT cell viability assay

4T1 cells were seeded in 96-well plates at a density of 10^4^ cells/well and were left to grow at 37°C for 72 h. After the incubation time, the medium was removed, and cells were washed three times with PBS, 100 µl of MTT solution (5 mg/ml; Research Products International, Mount Prospect, IL, M92050) was added to each of the wells and the plates were further incubated for another 4 h. After the incubation period, the MTT solution was removed from the wells, and 200 µl of MTT solubilization solution (10% Triton X-100, 80% isopropanol or 10.5 M) was added to solubilize the resulting crystals. The plate was covered with foil and placed on an orbital shaker for 10 min until all crystals were dissolved. The absorbance of each well was measured using a microplate reader (Biotek Instruments, Winooski, VT) at 570 nm. Each experiment had three technical replicates and was repeated at least five times. Statistical analysis was conducted using two-tailed unpaired Student's *t*-tests.

### *N*-acetyl-L-cysteine treatment

Cells were treated with 10 mM of NAC (Sigma-Aldrich, St. Louis, MO, A9165) for 1 h, transferred to ice, and immediately lysed using EB buffer.

## Supplementary Material

10.1242/joces.260104_sup1 Supplementary information
