# Supplementary material for: Vinculin Y822 is an important determinant of ligand binding
Source: J Cell Sci. 2023 Jun 14;136(12):jcs260104. doi: 10.1242/jcs.260104 (PMC10281268; doi:10.1242/jcs.260104)
Supplement: Supplementary information [file joces-136-260104-s1.pdf]

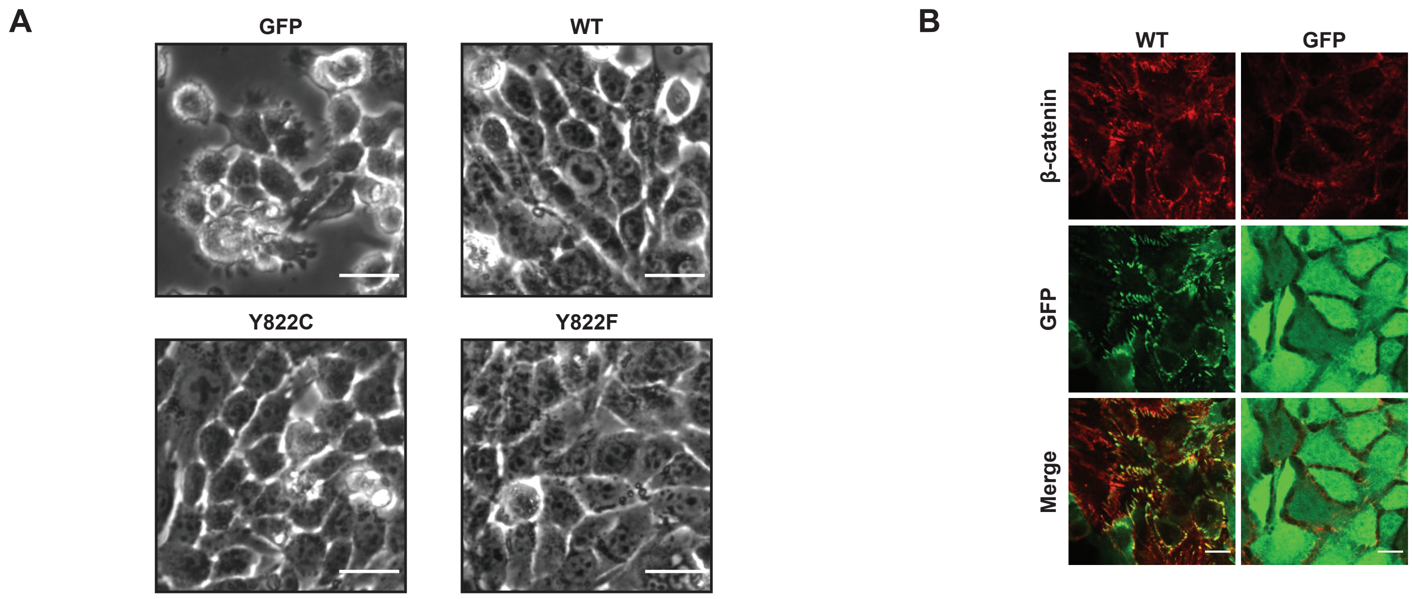

Fig. S1. The indicated cell lines do not form cell-cell adhesions

(A and B) The 4T1 cells pack closely with their neighbors but do not form cell-cell adhesions. **(A)** Higher magnification of phase images of the indicated 4T1 cells showing the apparent cell monolayers. Representative phase contrast images of indicated cells; scale bar, 30 μm. **(B)** 4T1 cells formed weak, punctated cell-cell adhesions. Confluent cultures of cells expressing GFP-wildtype (WT) or GFP were fixed, stained, and examined by immunofluorescence using an antibody against β-catenin. Representative images are shown. Scale bar, 10 μm.

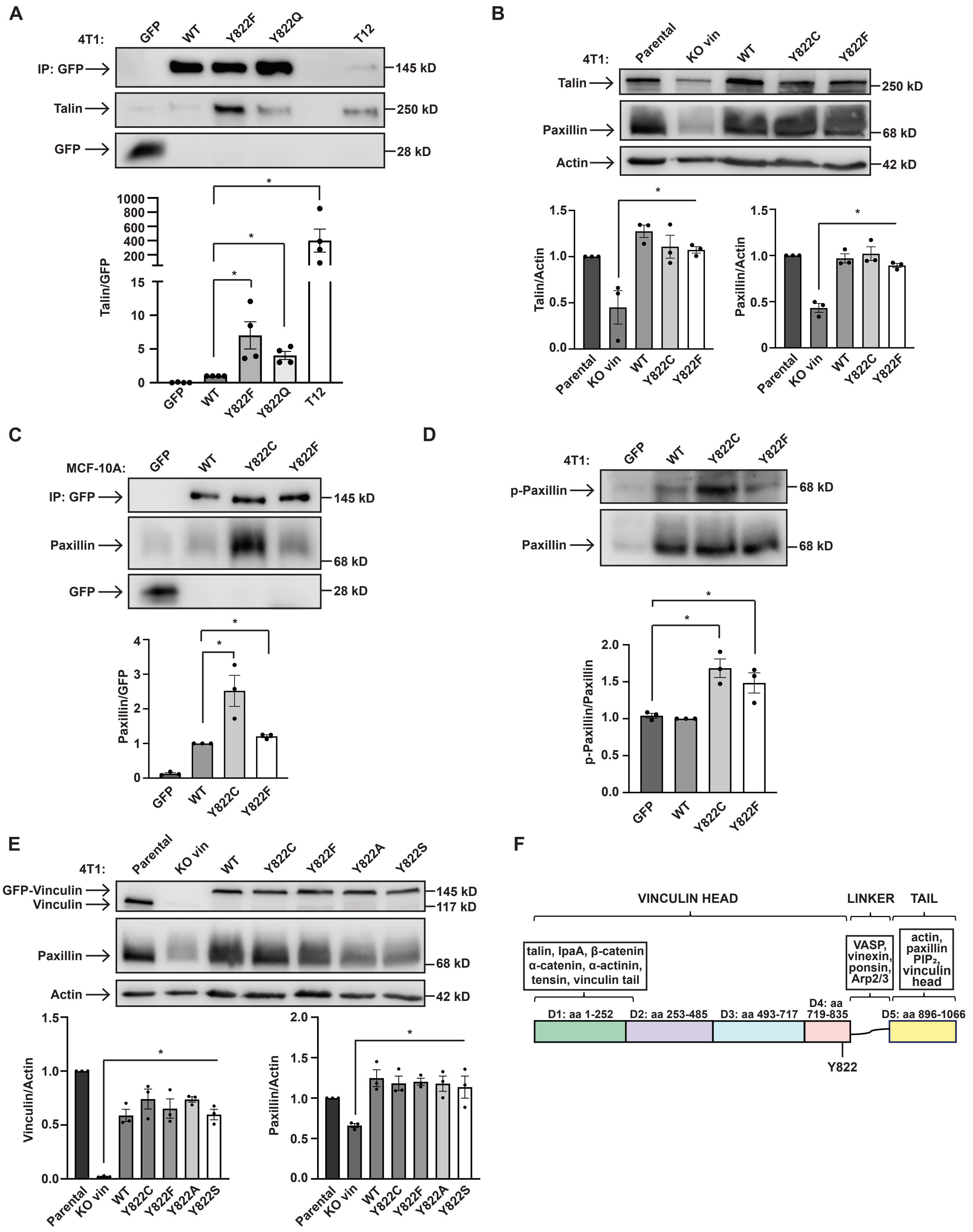

Journal of Cell Science • Supplementary information

Fig. S2. Additional characterization of the GFP-mutant vinculins and their binding to ligands

(A) Y822F vinculin does not bind talin to the same extent as T12 vinculin. The indicated GFP proteins were immunoprecipitated from cell lysates, and the co-precipitating levels of talin were examined by immunoblotting. The T12 vinculin binds talin so well that only half the amount of the immunoprecipitate was examined. The graph beneath the representative blots depicts the amount of talin recovered as a function of the amount of the GFP-protein in the sample. Data are mean ± s.e.m., $n = 4$ biologically independent samples. **(B)** Expression of indicated GFP-vinculin proteins rescued talin and paxillin protein expression. The levels of talin and paxillin were examined by immunoblotting whole cell lysates with antibodies against each respective protein with β-actin as a loading control. Data are mean ± s.e.m., $n = 3$ biologically independent samples. **(C)** Increased Y822C binding to paxillin was not cell type specific. The indicated GFP fusion proteins were stably expressed and then immunoprecipitated from MCF-10A cells. Talin co-precipitation was examined by immunoblotting. The graph beneath the blots represents the amount of paxillin bound normalized for GFP fusion protein expression. Data are mean ± s.e.m., $n = 3$ biologically independent samples. **(D)** Total phosphorylated paxillin is the same in all the cell lines analyzed. Lysates from the indicated cell lines were probed with antibodies that recognize phosphorylated (p-paxillin) and then stripped and re-probed for total paxillin levels. The graphs beneath the blots indicate the amount of phosphorylated paxillin in the samples as a function of the total paxillin levels. **(E)** Y822A and Y822S vinculins were expressed at the same level as the other vinculin fusion proteins. Lysates from the indicated cell lines were probed with antibodies against vinculin, paxillin and actin. The levels of vinculin or paxillin as a function of actin levels are shown. Data are mean ± s.e.m., n = 3 biologically independent samples. *, $p < 0.05$ by two-tailed unpaired Student's *t*-test.

Journal of Cell Science • Supplementary information

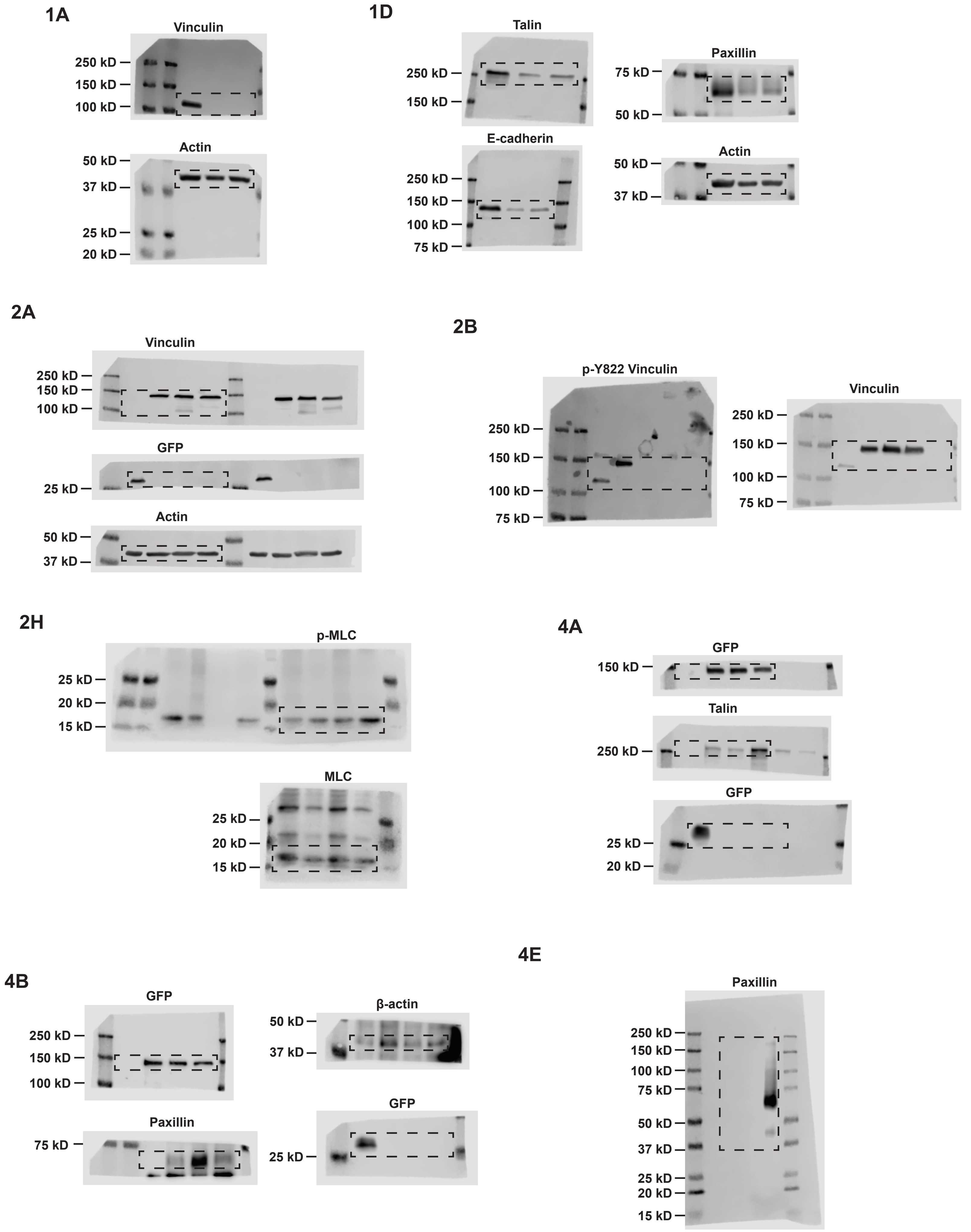

Fig. S3. Uncropped blots for figures 1, 2, and 4A-E.

The uncropped blots are shown. The dotted region indicates the portion of the blot included in the figures.

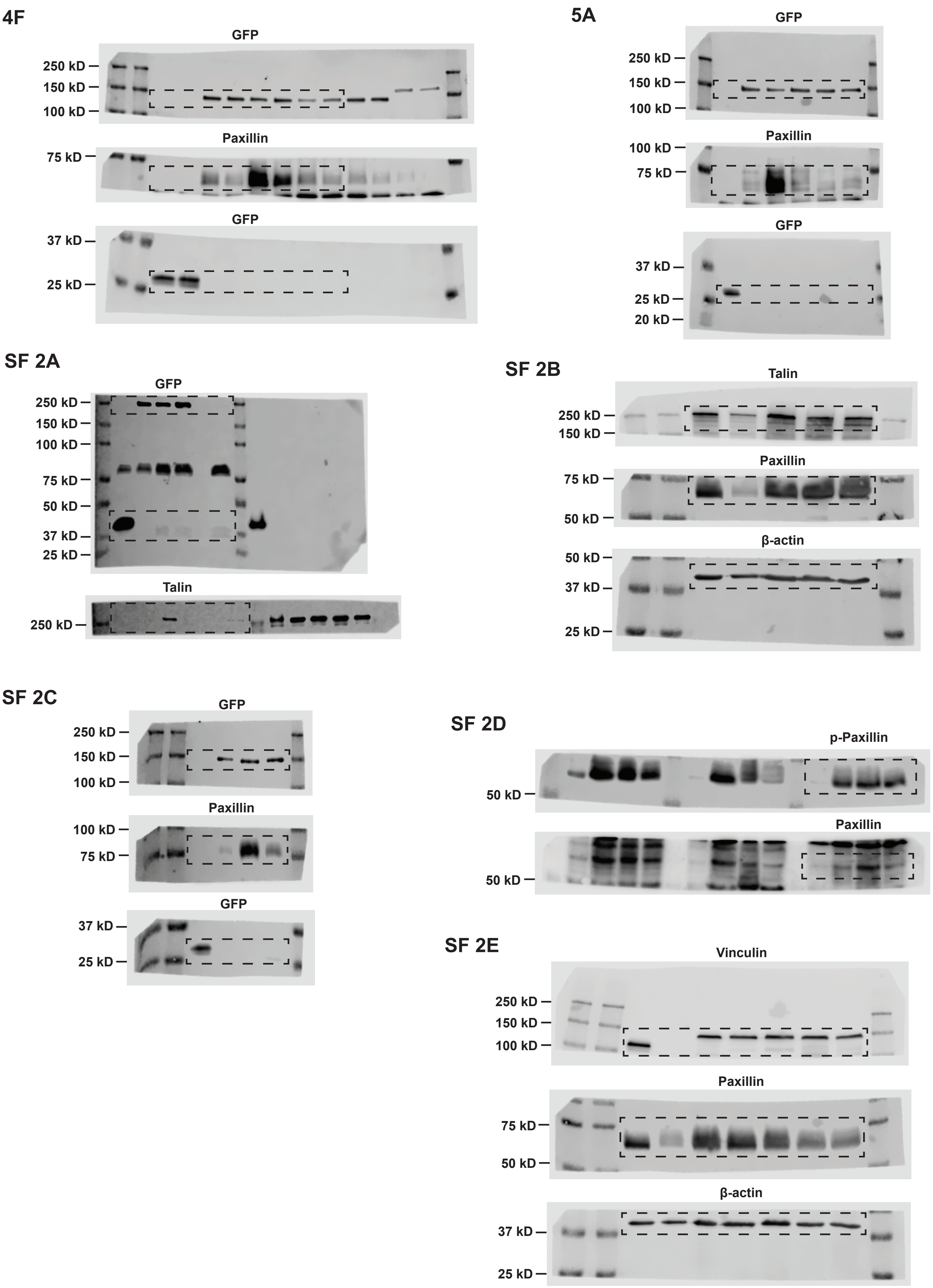

Fig. S4. Uncropped blots for figures 4F, 5 and supplemental figure 2. The uncropped blots are shown. The dotted region indicates the portion of the blot included in the figures.

Journal of Cell Science • Supplementary information
